# Gastroduodenal Artery Pseudoaneurysm Causing Obstructive Jaundice

**DOI:** 10.31486/toj.19.0110

**Published:** 2021

**Authors:** Brett M. Chapman, John S. Bolton, Scott M. Gioe, W. Charles Conway

**Affiliations:** ^1^ Department of Surgery, Ochsner Clinic Foundation, New Orleans, LA; ^2^ Department of Gastroenterology, Memorial Hospital, Gulfport, MS; ^3^ Department of Surgery, Ridley-Tree Cancer Center, Santa Barbara, CA

**Keywords:** *Aneurysm*, *aneurysm–false*, *jaundice–obstructive*, *pancreatitis*

## Abstract

**Background:** Visceral artery aneurysms and pseudoaneurysms are uncommon phenomena with a high mortality rate in cases of rupture. These rare vascular pathologies are usually asymptomatic and are therefore generally discovered incidentally on computed tomography or magnetic resonance imaging examination. Current therapeutic options have trended toward a minimally invasive approach because of evolving endovascular treatment options, with open operations typically reserved for cases of intraabdominal hemorrhage.

**Case Report:** We describe a case of gastroduodenal artery pseudoaneurysm manifesting as obstructive jaundice and pancreatitis because of extrahepatic compression of the common bile duct and pancreatic duct by mass effect. Open repair was ultimately required secondary to arterial anatomy that was not amenable to any endovascular treatment approach.

**Conclusion:** While endovascular options are the preferred treatment modality for visceral artery aneurysms and pseudo-aneurysms, some cases require definitive open repair for a variety of reasons, including unsuitable anatomy.

## INTRODUCTION

Visceral artery aneurysms and pseudoaneurysms are uncommon sequelae of pancreatitis, either acute or chronic, and are associated with significant morbidity and mortality because of their potentially devastating complications, with the most life-threatening complication being hemorrhage.^1,2^ Historically, open repair has been the gold standard in management.^3^ Since 2000, however, endovascular repair has become increasingly more common and has supplanted the open approach in the treatment algorithm when the patient is not in extremis and the patient's anatomy is amenable to a minimally invasive approach.^4^

We report the case of a patient with obstructive jaundice secondary to a gastroduodenal artery (GDA) pseudoaneurysm who ultimately required laparotomy, ligation of the GDA and anterior inferior pancreaticoduodenal artery, and aneurysmorrhaphy because of complex arterial anatomy that was not suitable for repair with endovascular techniques.

## CASE REPORT

A 74-year-old homeless male with a history of chronic alcoholic pancreatitis developed progressive epigastric abdominal pain and nausea that prompted his presentation to the emergency department at a community hospital. Laboratory studies showed mild transaminitis and hyperbilirubinemia (total bilirubin of 4.2 mg/dL) on initial evaluation. Computed tomography (CT) of the abdomen and pelvis revealed obstruction of the pancreatic and common bile ducts by what was felt to be a pancreatic head mass vs pseudoaneurysm of the GDA. Further evaluation with magnetic resonance cholangiopancreatography showed a 6-cm dilation of the proximal GDA consistent with the diagnosis of visceral artery pseudoaneurysm. Endoscopic retrograde cholangiopancreatography was performed with sphincterotomy and biliary stent placement. Pancreatic stent placement was also attempted but was unsuccessful because of significant external compression from the pseudoaneurysm that prevented cannulation of the major duct.

**Figure 1.**
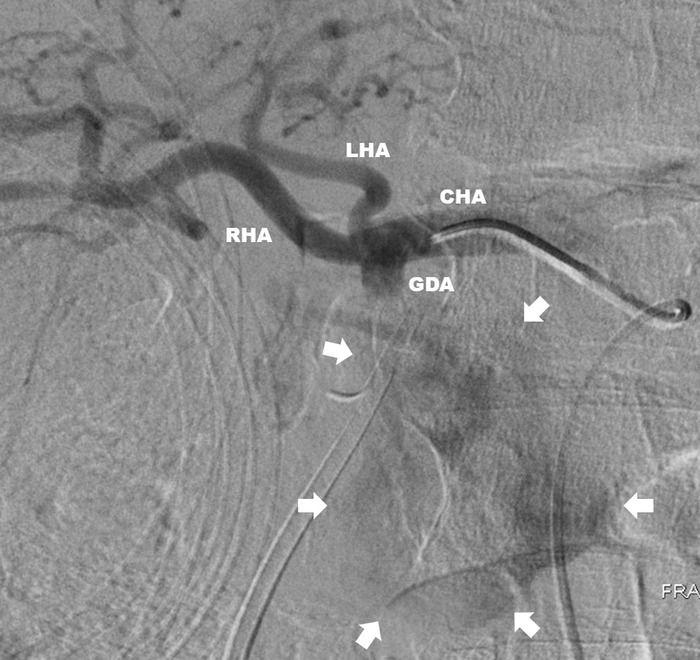
**Visceral angiography showing the gastroduodenal artery (GDA) pseudoaneurysm (arrows) and the complex anatomy that made it unsuitable for endovascular repair. Contrast was quickly dissipated because of the size of the pseudoaneurysm and the high-volume flow through it.** CHA, common hepatic artery; LHA, left hepatic artery; RHA, right hepatic artery.

**Figure 2.**
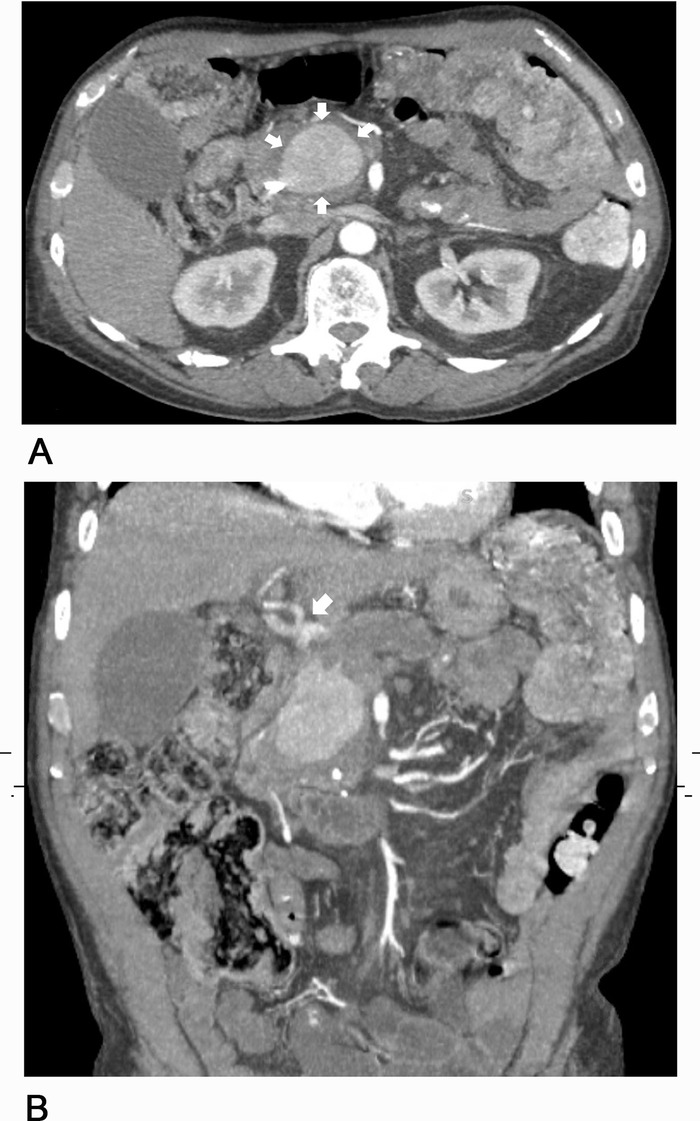
**Computed tomography angiography showing (A) axial cut of gastroduodenal artery pseudoaneurysm measuring 3.8 × 4.9 × 5.4 cm (arrows) and (B) coronal cut with arrow indicating trifurcation of the common hepatic artery.**

Following the patient's transfer to our facility for a higher level of care, interventional radiology was consulted for visceral angiography and possible coil embolization of the pseudoaneurysm. The angiographic anatomy (Figure 1) was deemed unsuitable for a variety of endovascular interventions. CT angiography was obtained to facilitate thorough operative planning for definitive repair (Figure 2).

After review of all available imaging, the patient was taken to the operating room. The abdomen was entered via an upper midline laparotomy. A Bookwalter self-retaining retractor (Symmetry Surgical, Inc.) was placed to provide wide exposure. The hepatic flexure and transverse colon were mobilized and retracted inferiorly away from the head of the pancreas. The pulsatile GDA pseudoaneurysm was easily palpable at this point. The gastrohepatic ligament was opened, and the common hepatic artery proximal to the GDA was mobilized. The proximal right and left hepatic arteries were mobilized and controlled with vessel loops given the lack of a proper hepatic artery between the GDA origin and the bifurcation. The pylorus and first portion of the duodenum were then mobilized away from the head of the pancreas. The liver was noted to have good arterial inflow from the celiac trunk, both on palpation and intraoperative ultrasound with temporary occlusion of the GDA. Clamps were applied proximally and distally following heparinization, and the GDA was divided at its origin. At this point, the surgical team believed that primary closure of the arteriotomy created by division of the GDA would result in potentially hemodynamically significant stenosis and compromise hepatic artery inflow. Bovine pericardial patch angioplasty was performed, and the proximal and distal clamps were released without evidence of compromised inflow. Examination of the pseudoaneurysm revealed some continued faint pulsatility. The duodenum was further mobilized, and the anterior inferior pancreaticoduodenal artery distal to the pseudoaneurysm was identified. The artery was clamped with cessation of all pulsatility and subsequently ligated. The pseudoaneurysm was then opened, a large amount of thrombus was evacuated, and an aneurysmorrhaphy was performed. A Blake drain was placed, and the abdomen was closed in the typical fashion to complete the case.

The abdominal drain was removed on postoperative day 7. The patient's postoperative course was uncomplicated aside from extensive social work issues because of his transient social situation and difficulty with posthospitalization placement.

## DISCUSSION

Pseudoaneurysms consist of a single layer of fibrous tissue around a sac of turbulent blood flow. These false aneurysms are the result of arterial wall disruption as a result of trauma, inflammation (eg, pancreatitis, autoimmune disorders), infection, or iatrogenic causes. As a result of normal arterial blood pressure, intraluminal blood leaks via this arterial wall disruption into the surrounding tissue to form a pseudoaneurysm sac that communicates directly with the arterial lumen.^4^ GDA pseudoaneurysm formation is an uncommon entity, and extrahepatic biliary compression causing obstructive jaundice is exceedingly rare.

One common cause of vessel injury, as with our patient, is severe pancreatic inflammation. Release of exocrine proteolytic and lipolytic enzyme-rich fluids into the peripancreatic space occurs as a result. Autodigestion of arterial walls by these enzymes and the resulting arteritis cause vessel wall architectural destruction and facilitate pseudoaneurysm formation.^1^

The reported incidence of pseudoaneurysm formation from pancreatitis varies from 1.3% to 10% among different case series.^5^ More than 3,000 cases of visceral artery aneurysms and pseudoaneurysms have been described in the literature.^1^ The distribution of splenic (60%), hepatic (20%), superior mesenteric (5.5%), celiac (4%), gastric and gastroepiploic (4%), intestinal (jejunal, ileal, colic) (3%), pancreatic and pancreaticoduodenal (2%), gastroduodenal (1.5%), and inferior mesenteric (rare) artery aneurysms and pseudoaneurysms has remained relatively constant since first investigated in 1996.^1,6,7^ The widespread availability and use of advanced imaging including CT, magnetic resonance imaging, ultrasound, and arteriography have led to the increased incidental detection of asymptomatic visceral artery aneurysms and pseudoaneurysms.^8^ Development of advanced endoscopic and endovascular techniques with instrumentation of the biliary tract and splanchnic vasculature has also increased the incidence of iatrogenic pseudoaneurysms.^9^

The management of pseudoaneurysms in the setting of pancreatitis can be challenging because of the risk of rupture and the potential for rapid and profound hemodynamic compromise if rupture occurs, all confounded by the morbidity of pancreatitis itself. The natural history of visceral artery pseudoaneurysms is not well defined. Reported mortality rates after rupture vary from 21% to 100% depending on location.^8,10^ Evidence suggests that pseudoaneurysms display the potential for relatively rapid growth rates in the immediate period following the initial insult, which underscores the importance of early diagnosis and early intervention regardless of size, location, or symptoms.^11^ In essence, pseudoaneurysms represent a contained rupture surrounded by only a fibrous layer, placing them at higher risk for overt rupture compared to true aneurysms. A 10-year retrospective review of 181 repaired aneurysms and pseudoaneurysms found that 81.8% of ruptures were pseudoaneurysms compared to 35.3% of intact repairs.^8^ Another 10-year retrospective review found that the rate of rupture was 76.3% with pseudoaneurysms vs 3.1% with true aneurysms.^12^

The goal of treatment consists of exclusion of the aneurysmal sac from the systemic circulation. This exclusion can be accomplished by either surgical or endovascular methods. Endovascular treatment of incidentally intact pseudoaneurysms has become the favored approach, with an expanding arsenal of techniques such as coil embolization, covered stent placement, endoluminal thrombin injection, plug deployment, and gelfoam embolization available to the interventional radiologist or vascular surgeon.^3,4,11,13^ In a retrospective case series from the Mayo Clinic reviewing the 10-year experience from 1999-2009 with visceral artery aneurysms (n=67) and pseudoaneurysms (n=118), 100% of cases were successfully treated with endovascular methods, highlighting the efficacy of minimally invasive techniques.^14^ Endovascular approaches are not without their own potential complications, however, and the most severe is an incompletely excluded aneurysm that remains at risk of rupture. Classically, surgical management involved open exposure to excise the aneurysm, with the specific situation dictating the necessity of reestablishment of vascular continuity or end organ resection.^3,15^ As one would expect, surgical access to these sites in a hostile environment carries the potential for significant technical difficulty, as well as morbidity and mortality. Operative treatment is now largely reserved for patients who are hemodynamically unstable; fail repeat coil embolization with continued or recurrent bleeding; or have other indications such as pancreatic abscess, pseudocyst, gastric outlet obstruction, or obstructive jaundice not amenable to other available interventions.

In our patient, a large, angulated pseudoaneurysm with complex arterial anatomy precluded multiple endovascular interventions. The short, large-diameter GDA origin eliminated coil embolization and covered stent options because of the inability to land coils or a stent in the proximal GDA without occluding hepatic artery inflow. Anomalous arterial anatomy with trifurcation of the GDA, right hepatic, and left hepatic without a discernable proper hepatic artery also eliminated a hepatic artery stent with distal coiling of the pseudoaneurysm as an option (Figures 1 and 2). Additionally, these endovascular solutions would provide little in terms of short-term relief of the ongoing obstruction, as the pseudoaneurysm would require several days to weeks to thrombose and decrease in size to adequately relieve the obstruction without pancreatic stent drainage. Our patient is one of only a handful of cases identified in our review of the literature to present with a GDA aneurysm or pseudoaneurysm causing biliary obstruction and even fewer to require an open operation.^16-20^

## CONCLUSION

Operative repair of visceral artery pseudoaneurysms remains a viable option when the anatomy is prohibitive for endovascular intervention but is not without considerable risk.

## Notes

This article meets the Accreditation Council for Graduate Medical Education and the American Board of Medical Specialties Maintenance of Certification competencies for Patient Care and Medical Knowledge.
